# Single-cell RNA sequencing reveals the mechanism of sonodynamic therapy combined with a RAS inhibitor in the setting of hepatocellular carcinoma

**DOI:** 10.1186/s12951-021-00923-3

**Published:** 2021-06-12

**Authors:** Bolin Wu, Yanchi Yuan, Jiayin Liu, Haitao Shang, Jing Dong, Xitian Liang, Dongxu Wang, Yichi Chen, Chunyue Wang, Yang Zhou, Hui Jing, Wen Cheng

**Affiliations:** 1 Department of Ultrasound, Harbin Medical University Cancer Hospital, No.150, Haping Road, Nangang District, Harbin, 150081 Heilongjiang China; 2 Department of Interventional Ultrasound, Harbin Medical University Cancer Hospital, Harbin, China; 3 Institute of Cancer Prevention and Treatment, Heilongjiang Academy of Medical Science, Harbin, China; 4 Department of Radiation Oncology, Harbin Medical University Cancer Hospital, Harbin, China; 5 Department of Radiology, Harbin Medical University Cancer Hospital, Harbin, China

**Keywords:** Hepatocellular carcinoma, Sonodynamic therapy, scRNA-seq, RAS inhibitor

## Abstract

**Background:**

Ras activation is a frequent event in hepatocellular carcinoma (HCC). Combining a RAS inhibitor with traditional clinical therapeutics might be hampered by a variety of side effects, thus hindering further clinical translation. Herein, we report on integrating an IR820 nanocapsule-augmented sonodynamic therapy (SDT) with the RAS inhibitor farnesyl-thiosalicylic acid (FTS). Using cellular and tumor models, we demonstrate that combined nanocapsule-augmented SDT with FTS induces an anti-tumor effect, which not only inhibits tumor progression, and enables fluorescence imaging. To dissect the mechanism of a combined tumoricidal therapeutic strategy, we investigated the scRNA-seq transcriptional profiles of an HCC xenograft following treatment.

**Results:**

Integrative single-cell analysis identified several clusters that defined many corresponding differentially expressed genes, which provided a global view of cellular heterogeneity in HCC after combined SDT/FTS treatment. We conclude that the combination treatment suppressed HCC, and did so by inhibiting endothelial cells and a modulated immunity. Moreover, hepatic stellate secretes hepatocyte growth factor, which plays a key role in treating SDT combined FTS. By contrast, enrichment analysis estimated the functional roles of differentially expressed genes. The Gene Ontology terms “cadherin binding” and “cell adhesion molecule binding” and KEGG pathway “pathway in cancer” were significantly enriched by differentially expressed genes after combined SDT/FTS therapy.

**Conclusions:**

Thus, some undefined mechanisms were revealed by scRNA-seq analysis. This report provides a novel proof-of-concept for combinatorial HCC-targeted therapeutics that is based on a non-invasive anti-tumor therapeutic strategy and a RAS inhibitor.

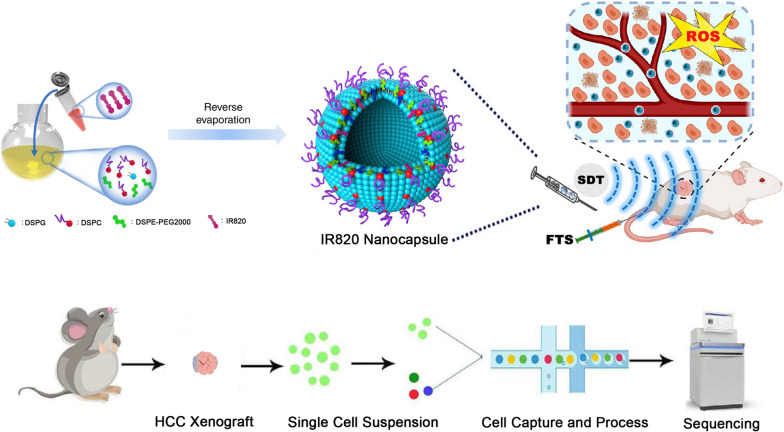

**Supplementary Information:**

The online version contains supplementary material available at 10.1186/s12951-021-00923-3.

## Background

Worldwide, liver cancer is the fourth most common cause of cancer-related death and correlates with a poor prognosis [1]. With a 5-year survival rate of only 18%, liver cancer is the second most lethal tumor after pancreatic cancer. Hepatocellular carcinoma (HCC) is the major form of primary liver cancer. More than 700,000 cases of HCC are diagnosed, and approximately half a million people die of liver cancer every year [2, 3]. Many treatments are currently available for HCC, including surgical resection, liver transplantation, chemotherapy, and radiotherapy [4, 5]. Indeed, a few patients have access to timely treatment since many of them are usually diagnosed with advanced liver cancer [2]. Hence, the discovery and development of innovative anti-HCC combinatorial therapies with lower host toxicity have evolved as a globally pressing task. Many nanomedicines have been invented for HCC and rodent hepatoma model treatment [6–8]. In addition, some nanocapsules could also promote chemotherapy and induce an immune response, which synergistically performed antitumor efficacy [9–11].

Activation of the Ras signaling pathway is a ubiquitous event in HCC. Ras signaling has been shown to contribute to the initiation of cancer cell development and the resistance of HCC cells to apoptosis [12]. The activated Ras cascade phosphorylates its downstream targets Raf, MEK, and Erk to regulate cell proliferation, differentiation, and cycling, which is a key oncogenic signal transduction pathway activated in HCC [13]. Targeting Ras is regarded as a potential approach to treating HCC [14]. Ras mutation plays a very important role in HCC. Both H-ras and K-ras have respective splice variants, which may provide a new approach to HCC therapy.

Farnesylthiosalicylic acid (FTS, salirasib) is an effective Ras inhibitor, which can dislodge Ras from its membrane anchoring sites and facilitate its degradation, thereby modulating the downstream signaling pathway of Ras and inhibiting Ras-dependent cancer cells growth [15, 16].

In 1989, Prof. Yumita first reported sonodynamic therapy (SDT) [17], which is based on photodynamic therapy (PDT). PDT could generate immunogenic cell death (ICD), accompanied by the release of high-mobility group box 1 protein (HMGB1) and adenosine triphosphate (ATP) and the exposure of calreticulin (CRT), sending the “eat me” signal and promoting the antigen presentation and maturation of dendritic cells (DCs) [18]. Also, Photothermal therapy (PTT) is a developing field that uses the strategy of employing near-infrared (NIR) irradiation to trigger drug delivery [19]. These emerging non-invasive techniques have been widely used in anti-tumor research [20–23]. SDT is already known as a potential anti-cancer strategy that uses non-thermal ultrasound energy combined with sonosensitizer agents [24–27]. Generally, low-intensity focused ultrasound (LIFU, 1–3 MHz) is used for this therapeutic technology to enhance the cavitation effect [28, 29]. Sonosensitizer agents combined with ultrasound irradiation generate reactive oxygen species (ROS) that have the potential of inducing cancer-cell death under aerobic conditions [30–32].

Several reports have shown that LIFU could enhance the drug sensitivity of some select chemotherapeutic drugs and could improve cell membrane permeability [33–35]. Our previous research showed that the combination of LIFU exposure and the nanobubble system can be used as an efficient and safe approach for gene delivery and transfection [36, 37]. Furthermore, LIFU combined NET-1 siRNA conjugated nanobubble complexes have been shown to slow down tumor growth and prolong the survival of experimental animals [38].

In the current study, we recognize that single-cell RNA sequencing (scRNA-seq) is a powerful tool with which to explore cellular heterogeneity [39–42]. In working with the 10× Genomics Chromium Single Cell Gene Expression workflow, we have studied cellular heterogeneity of HCC after combined SDT and FTS therapy.

## Results

### Formulation and characterization of the IR820 nanocapsule

**Fig. 1:**
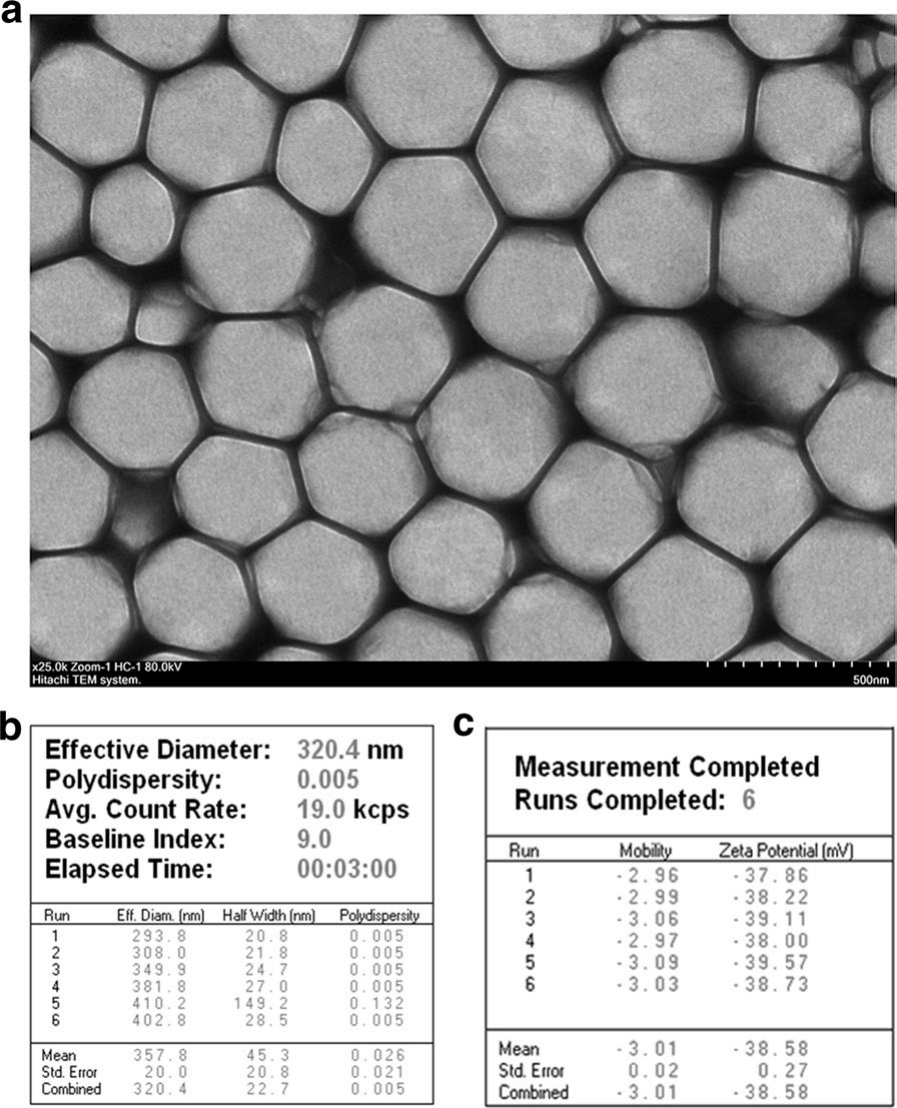
Structure and characterization of the composite IR820 nanocapsule. **a** Transmission electron microscopy image showing a quasi-spherical morphology of IR820@NCP with a mean diameter of about 300 nm and high dispersity. Original magnification =  ×25,000. Scale bar = 500 nm; **b** The DLS results showed a mean particle size of IR820@NCP to be 357.8 ± 20.0 nm with a 0.005 polydispersity value; and **c** The Zeta PALS BI-90 Plus analyzer indicated a surface Zeta potential of IR820@NCP to be − 38.58 ± 0.27 mV

The obtained IR820@NCP was well-dispersed in an aqueous solution and appeared as a smooth round surface under TEM (Fig. 1a). The mean diameter of IR820@NCP was 357.8 ± 20.0 nm, with a polydispersity value of 0.025 (Fig. 1b). Meanwhile, the zeta potential value of the complexes was − 38.58 ± 0.27 mV (Fig. 1c). The stability of IR820@NCP is expressed in concentration. The IR820@NCP concentration is diluted to 4 × 10^8^/ml. The concentrations of different times were shown in Additional file 1: Figure S1. Brown–Forsythe test shown the P value was 0.6917, which demonstrated no statistical significance in concentration changes within 48 h.

### Cytotoxicity of IR820 and FTS

We first used the CCK-8 assay to evaluate the cytotoxicity of IR820 and FTS, respectively. We found that when incubated with IR820 or FTS alone, dose-dependent cytotoxicity was induced. The influence on cell viability is revealed in Additional file 2: Figure S2a, b. The IC50 values were 3.497 μM for IR820 (Additional file 2: Figure S2c) and 169.6 μM for FTS (Additional file 2: Figure S2d).

### Synergistic curative effect of combined SDT and FTS treatment in vitro

**Fig. 2:**
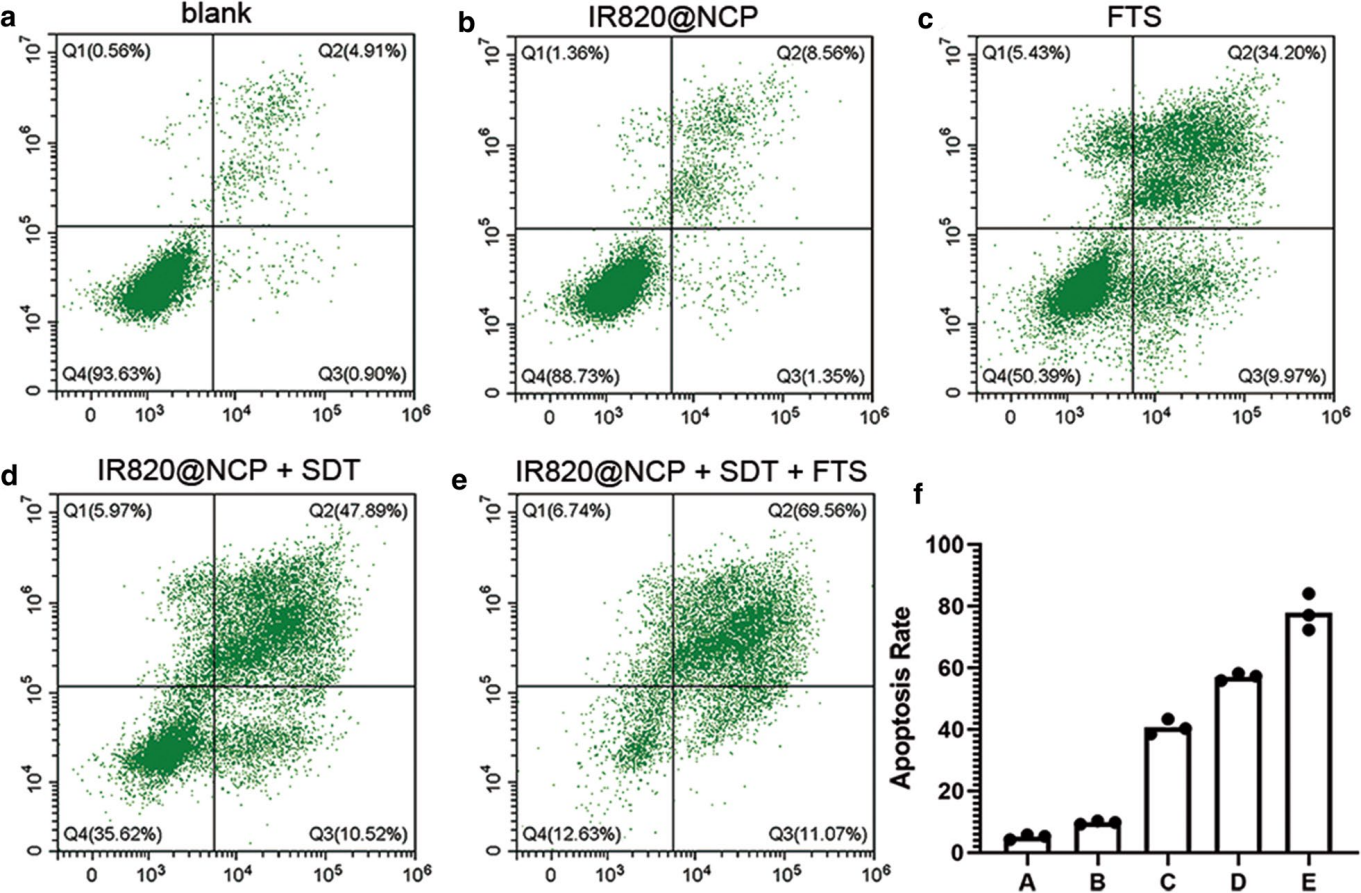
In vitro curative effect of the IR820 nanocapsule upon SDT and FTS. **a**–**e** The apoptotic frequency was analyzed by flow cytometry after different treatments. The total apoptosis rate was calculated by Q2 (early apoptosis) and Q3 (late apoptosis). **f** Quantification of the total apoptosis rates. Data are expressed as mean ± SD (n = 3). Statistical significances were calculated via one-way analysis of variance (ANOVA)

The curative effect of IR820@NCP and FTS against cancer cells was evaluated by flow cytometry. The representative dot-plots illustrating apoptotic status are shown in Fig. 2a–e, and the corresponding statistical results from three independent experiments are shown in Fig. 2f. Statistical analysis showed that the frequency of apoptotic cells that were induced by various treatments increased from 5.1 ± 0.82% (blank) and 9.8 ± 0.55% (IR820@NCP) to 40.7 ± 2.5% (FTS), 57.1 ± 1.16% (IR820@NCP + SDT) and 77.9 ± 5.89% (IR820@NCP + SDT + FTS) in HepG2 cells (P < 0.05).

### Analysis of ROS and MMP

**Fig. 3:**
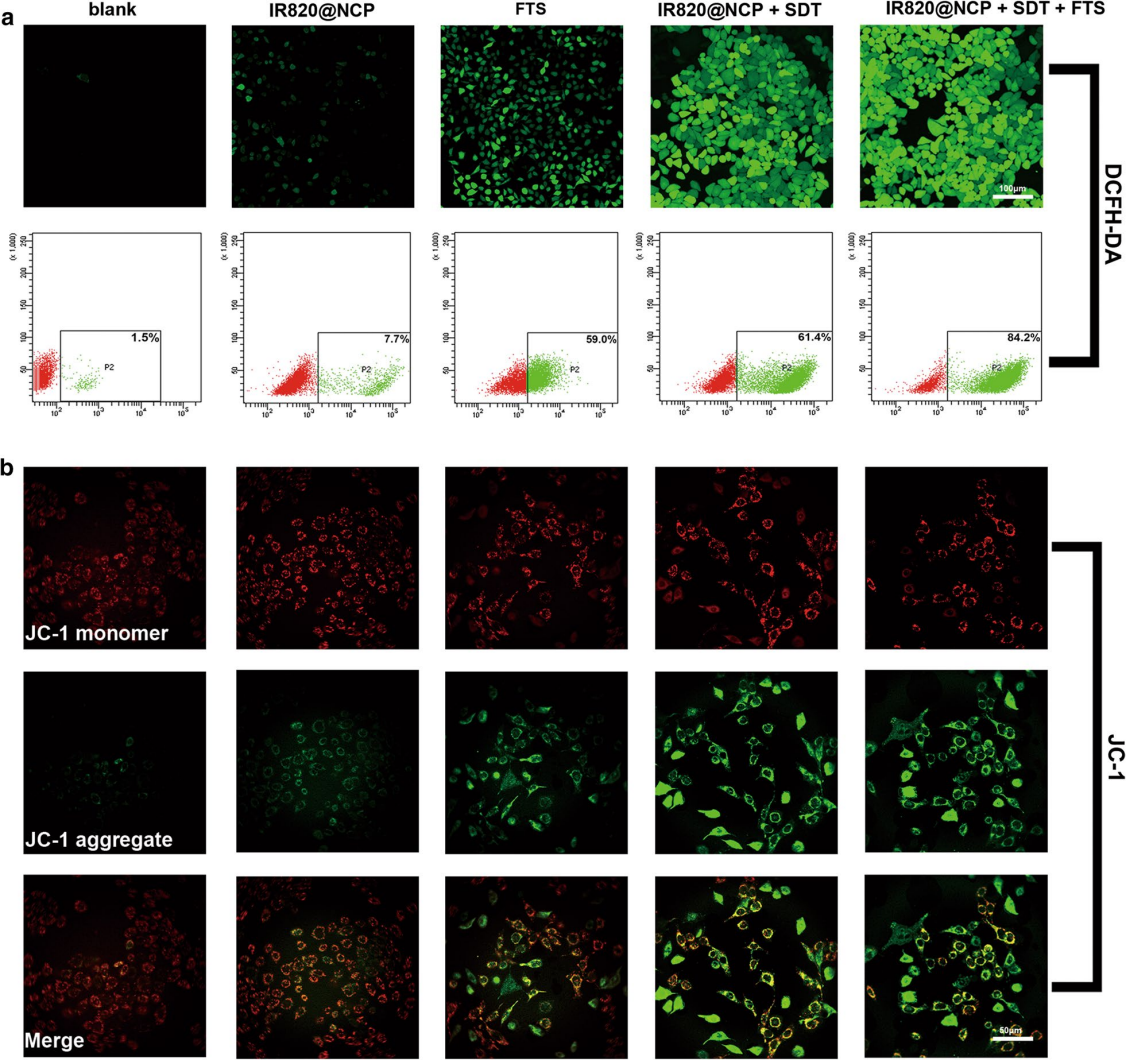
Analysis of ROS and MMP. **a** CLSM images and flow cytometry analyses of intracellular ROS generation as indicated by DCFH-DA detection after receiving different treatments as indicated. **b** CLSM images of the JC-1 monomer (green channel), and aggregate (red channel) in the mitochondria of HCC cells after differential treatments as indicated

Reactive oxygen species (ROS) generation was measured with the oxidation-sensitive probe DCFH-DA. As expected, both IR820@NCP + SDT and IR820@NCP + SDT + FTS intracellularly provoked high levels of ROS after treatment, as shown by intense intracellular green fluorescence in HepG2 cells (Fig. 3a). Representative dot-plots illustrating ROS are shown in Fig. 3a (P < 0.001).

Depolarization of the mitochondrial membrane resulting in a loss of MMP is a universal event during the intrinsic apoptotic pathway [43]. SDT has been proved to induce the loss of MMP, resulting in cell death [44, 45]. As shown in Fig. 3b, Group A and Group B cells mainly exhibited a red fluorescence. Comparatively, FTS-treated cells exhibited a green fluorescence with sporadic red fluorescence, indicating that FTS induces a slight loss in MMP. The IR820@NCP + SDT group showed more green fluorescence than the FTS group, which indicated that SDT can induce stronger loss of MMP than FTS. Further, FTS pretreatment combined with IR820@NCP and SDT potently enhanced the green fluorescence of cells, indicating that SDT significantly increased FTS-induced loss of MMP.

### Multi-wavelength fluorescence imaging

**Fig. 4:**
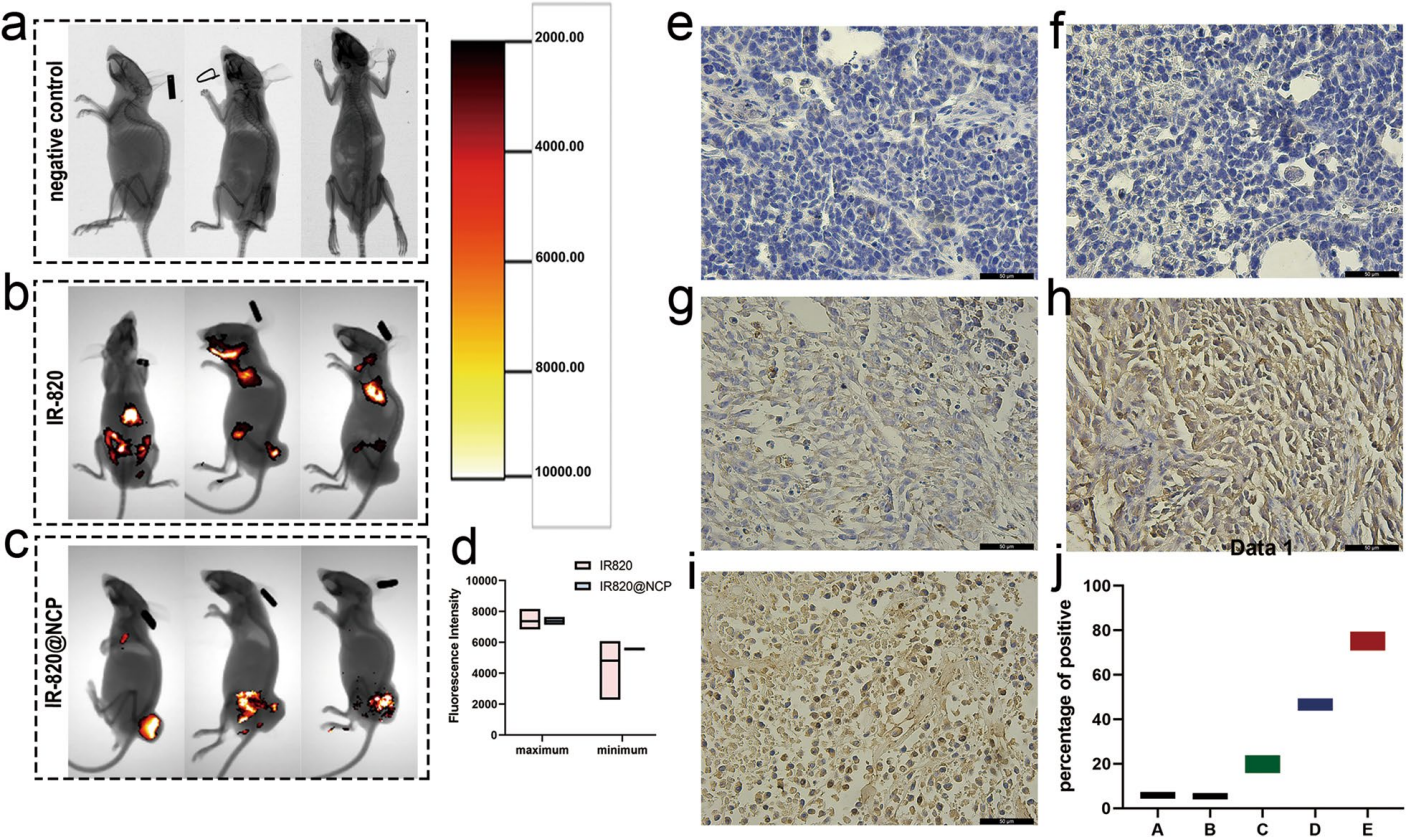
In vivo Multi-wavelength Fluorescence Imaging analysis and anti-tumor efficiency. **a**–**c** In vivo multi-wavelength fluorescence images tracking the spread of i.v. injected IR-820 and IR820@NCP in the mouse model. **d** Quantification of the maximum and minimum fluorescence intensities. **e**–**i** Example of the variability of caspase-3 staining within tumor samples in group A-E. **j** Quantitative analysis of all groups. Original magnification, ×400, Scale bar, 50 μm

To evaluate targeted tumor imaging of the IR820@NCP, a tumor xenograft model was initially explored with an in vivo fluorescence imaging system. For each nude mouse, two images were acquired: (1) an X-ray structural image, and (2) a multi-wavelength fluorescence image. Briefly, no fluorescence was detected in the negative control group (Fig. 4a). The mice in the IR820 group showed sporadic fluorescence in the body (Fig. 4b). By contrast, mice in the IR820@NCP group showed increased fluorescence within the tumor region, whereas fluorescence was absent from other bodily regions (Fig. 4c). Observations showed that IR820@NCP had the potential for targeted tumor imaging, due in part to the enhanced permeability and retention (EPR) effect. However, the maximum and minimum intensity between both groups were statistically non-significant (P = 0.4896, Fig. 4d).

On the other hand, no fluorescence was detected in the hearts, lungs, and spleens (Additional file 3: Figure S3a–c). However, kidneys showed faint fluorescence (Additional file 3: Figure S3d), which can be attributed to renal metabolism of IR820.

### Anti-tumor efficiency in vivo

The percentage of caspase-3 positive cells was quantified by ImageJ. Quantitative IHC analysis indicated that the caspase-3 positive staining was 5.68 ± 1.49 (group A, Fig. 4e), 5.33 ± 1.31 (group B, Fig. 4f), 20.02 ± 4.04 (group C, Fig. 4g), 46.58 ± 2.72 (group D, Fig. 4h), and 74.26 ± 4.45 (group E, Fig. 4i) compared with the whole picture in each group (Fig. 4j, P < 0.01, one-way ANOVA).

Nearly no positive immunostaining for caspase-3 was observed in group A and group B (Fig. 4e, f). However, the caspase-3 expression was significantly upregulated in the remaining three groups (Fig. 4g–i).

### Single-cell transcript coverage and representation

According to the scRNA-Seq data, we successfully sequenced 7283, 7610, 3724, and 3103 cells from Groups A through E, respectively (Additional file 6: Table S1). To characterize the cell types of HCC xenograft single cells, we grouped single cells into eleven clusters for Group A and C (Additional file 4: Figure S4a, b), and ten clusters for Group D and E were projected on the tSNE plot (Additional file 4: Figure S4c, d). Cluster identity was defined based on known cell-type markers and differentially expressed genes (Additional file 5: Figure S5).

### Integrative single-cell analysis

The aim here was to investigate the treatment mechanism of combined SDT and FTS therapy, and the cellular heterogeneity of HCC following differential treatments. The integrative single-cell analyses of Group A vs Group C; Group A vs Group D; and Group A vs Group E were conducted to obtain further explore the scRNA-seq data.

**Fig. 5:**
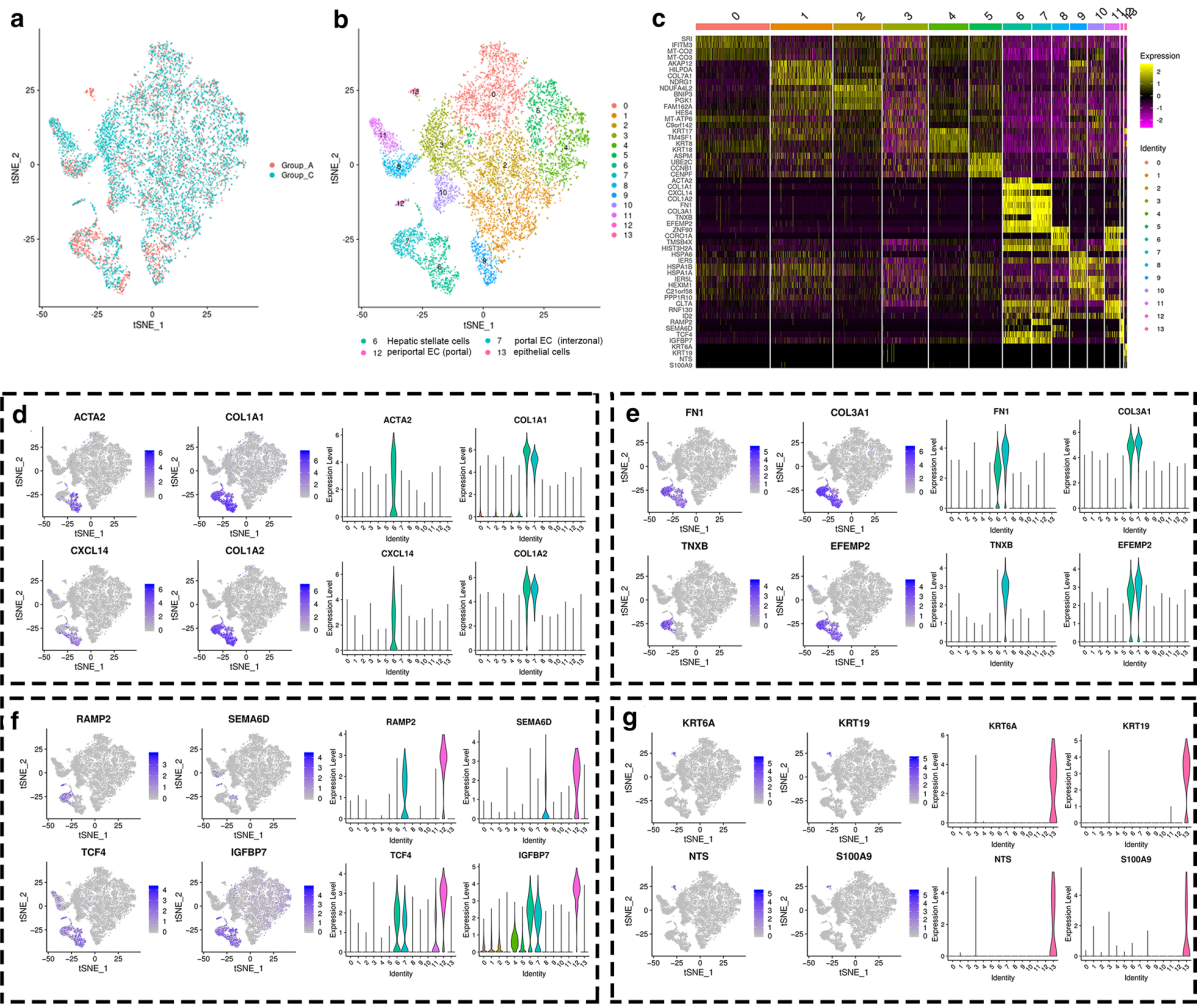
Integrative single-cell analysis of Group A and C. **a** tSNE plot of single-cell transcriptomic data with cells colored by Group A and Group C as indicated in the figure key. **b** Two-dimensional tSNE representation of single cells, in which cells were colored according to cluster identity as shown in the figure key. **c** Heatmap of highly differentially-expressed genes after FTS treatment

After integrative single-cell analysis of the negative control and FTS treatment groups (Fig. 5a), the cells were identified into thirteen clusters (Fig. 5b), which were based on differentially expressed genes (Fig. 5c). Among the clusters between Groups A and C, clusters 6, 7, 12, and 13 were significantly evolved. Cluster 6 represents hepatic stellate cell populations since many of the cells in this cluster express pluripotent genes like ACTA2, and CXCL14 (Fig. 5d). Cells in cluster 7 represent populations of interzonal portal endothelial cells due in large part to the expression of the TNXB gene (Fig. 5e). Also, cells in clusters 6 and 7 share the expression profile of several pluripotent genes, including COL1A1, COL1A2, FN1, COL3A1, and EFEMP2 (Fig. 5f). The cells in cluster 12 represent portal periportal endothelial cell populations since most cells in this cluster express IGFBP7 and SEMA6D genes (Fig. 5f). Cells in cluster 13 represent typical epithelial cells, while epithelial cell genes like KRT6A, KRT19, NTS, and S100A9 were significantly expressed (Fig. 5g).

**Fig. 6:**
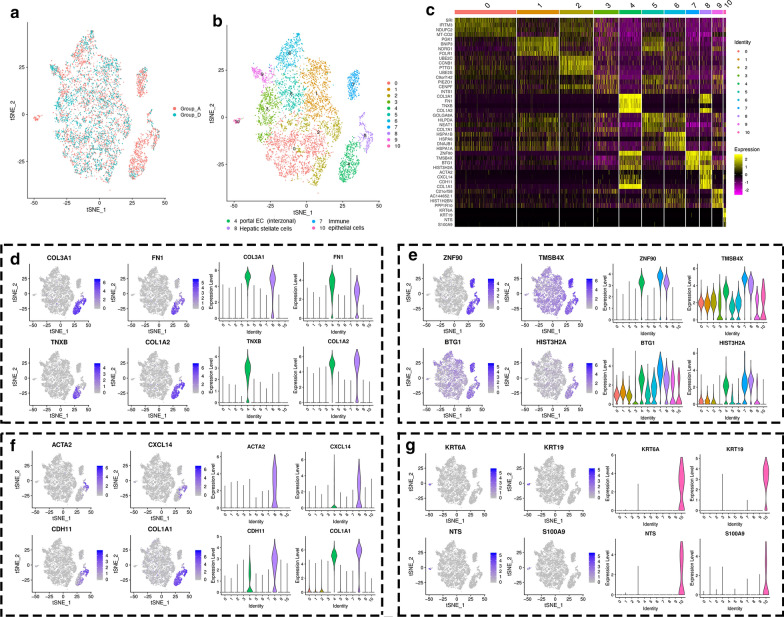
Integrative single-cell analysis of Group A and Group D. **a** tSNE plot of single-cell transcriptomic data with cells colored by Group A and Group C as indicated in the figure key. **b** Two-dimensional tSNE representation of single cells, in which cells were colored according to cluster identity as shown in the figure key. **c** Heatmap of highly differentially-expressed genes after FTS treatment

Comparing Groups A and D (Fig. 6a), the cells were segregated into ten clusters (Fig. 6b) following integrative single-cell analysis based on differentially expressed genes (Fig. 6c). Clusters 4, 7, 8, and 10 showed significant differences after sonodynamic therapy. The cells in clusters 4, 8, and 10 represent portal periportal endothelial cell populations, hepatic stellate cells, and typical epithelial cell populations as described above (Figs. 6d, f, g). The cells in cluster 7 represent immune-related cells since immune-associated genes like ZNF90, TMSB4X and BTG were highly expressed in this cluster (Fig. 6e). Besides, the cells also share the HIST3H2A gene with clusters 4 and 8.

**Fig. 7:**
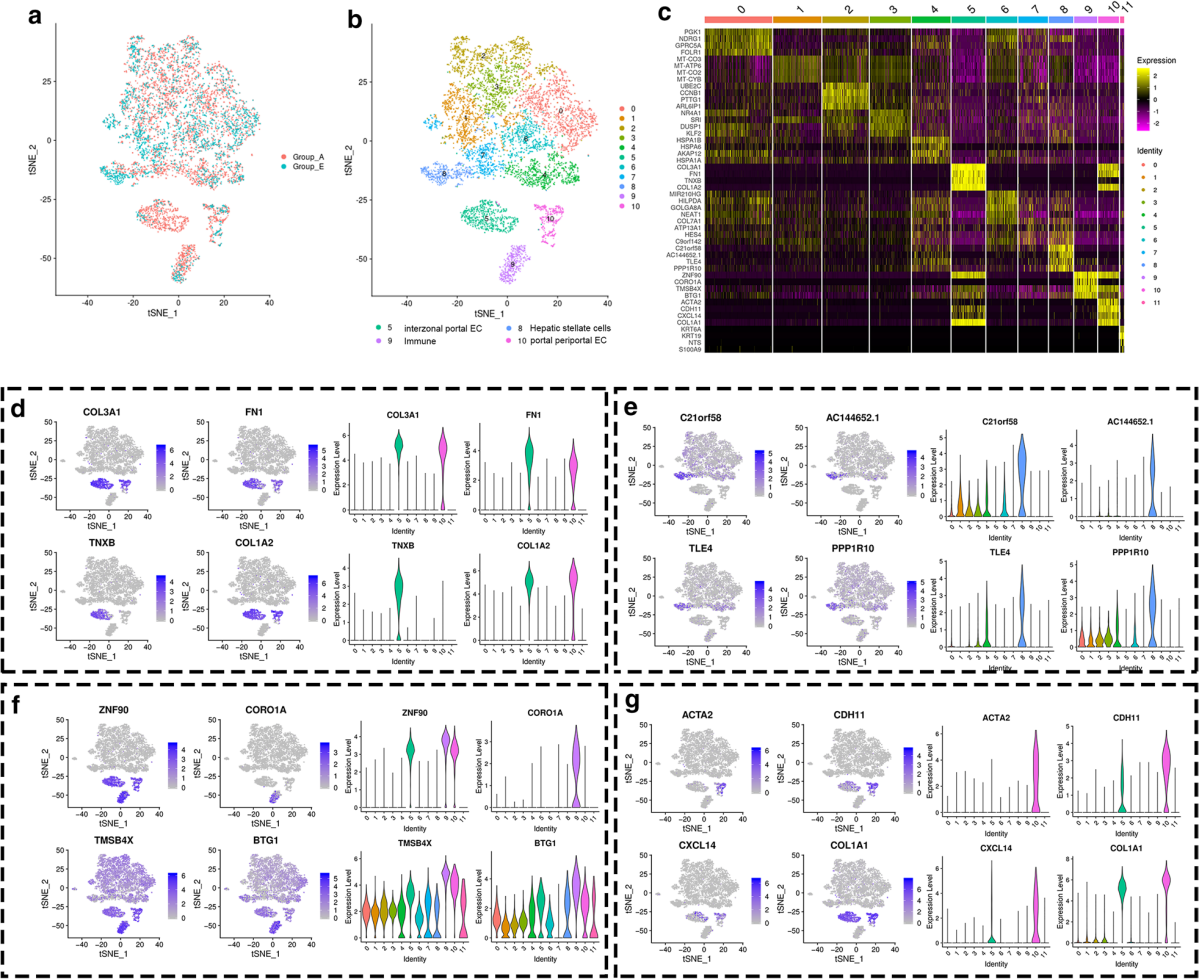
Integrative single-cell analysis of Group A and Group E. **a** tSNE plot of single-cell transcriptomic data with cells colored by Group A and Group E as indicated in the figure key. **b** Two-dimensional tSNE representation of single cells, in which cells were colored according to cluster identity as shown in the figure key. **c** Heatmap of highly differentially-expressed genes after the combined treatment of SDT and FTS

After integrative single-cell analysis of the negative control with the combined SDT and FTS treatment group (Fig. 7a), the cells were segregated into ten clusters (Fig. 7b) based on differentially expressed genes (Fig. 7c). Among the clusters between Groups A and E, clusters 5, 9, and 10 were significantly evolved. Similar to previous integrative single-cell analyses, the variant clusters were identified as inter-zonal portal endothelial cell populations (Fig. 7d), immune-related cell populations (Fig. 7f), and portal periportal endothelial cell populations (Fig. 7g) according to differentially expressed genes, respectively. Cluster 8 was likely to be a hepatic stellate cell population since the genes, including C21orf58, AC144652.1, TLE, and PPP1R10, were significantly and highly expressed in this cluster (Fig. 7e). Strangely, hepatic stellate cell populations expressed different genes after FTS and combined SDT and FTS treatment.

### Enrichment analysis of differentially expressed genes

**Fig. 8:**
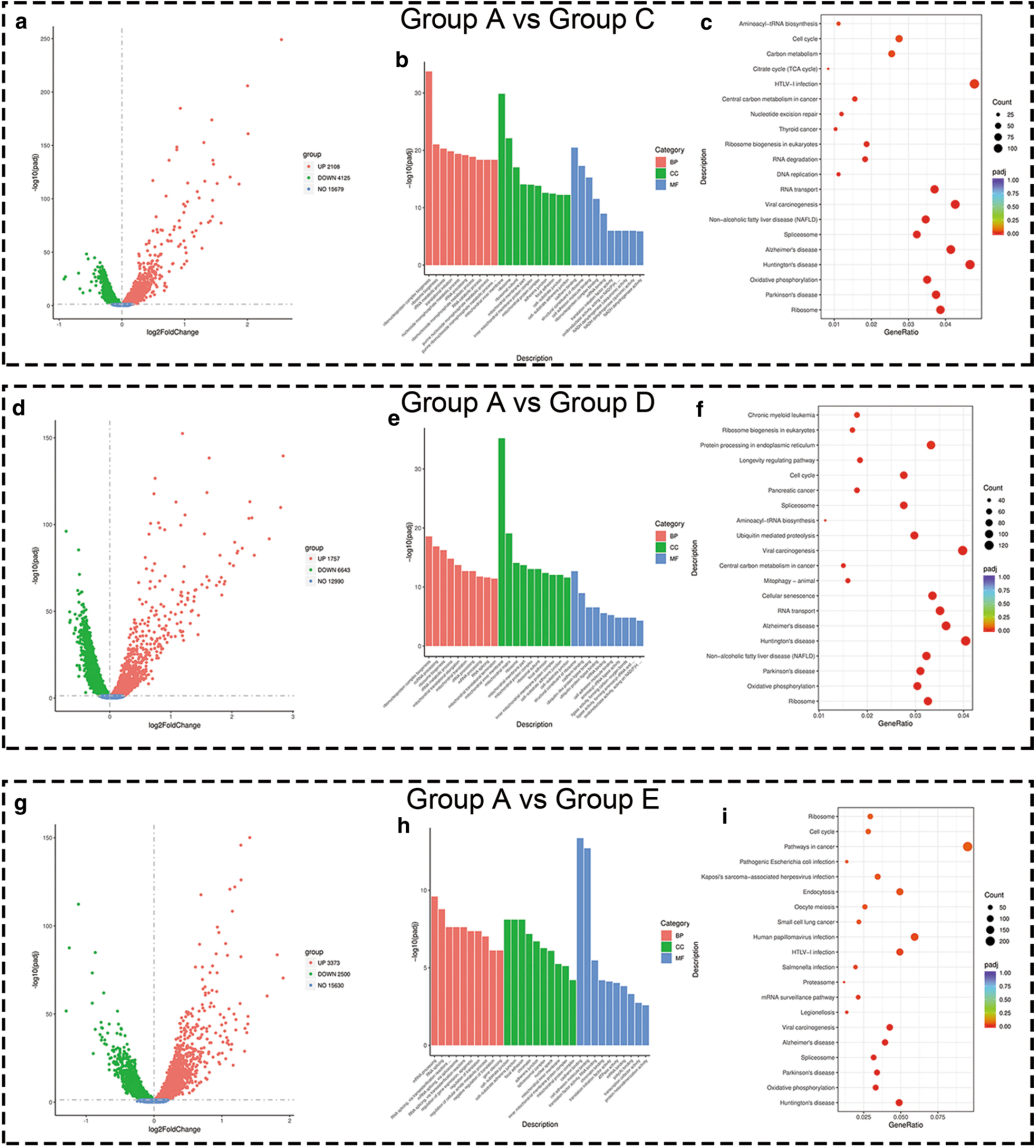
Enrichment results of differentially expressed genes. **a**, **d**, and **g** showing volcano plots of differentially-expressed genes on comparing group A with C, group A with D, and group A with E. Each red dot denotes an individually up-regulated gene, and each green dot denotes an individually down-regulated gene. **b**, **e**, and **h** Showing G.O. terms of differentially-expressed genes on comparing group A with C, group A with D and group A with E. Red columns denote the biological process terms, green columns denote the cellular component terms, and blue columns denote the molecular function terms (**c**, **f**, and **i**) showing the top 20 over-represented KEGG pathways on comparing group A with C, group A with D, and group A with E

Differential gene expression was estimated by expression levels (FPKMs). Consequently, it was found that 6233 genes were differentially expressed on comparing group A with C (P-adjust < 0.05), which included 2108 up-regulated genes and 4125 down-regulated genes (Fig. 8a). Based on the GO survey, 1266 differential GO terms showed significant enrichment in group A as compared to group C (Additional file 7: Table S2). Simultaneously, 23 pathways were enriched by differentially-expressed genes (Additional file 8: Table S3). The representative GO enriched terms and KEGG pathways of differentially expressed genes are respectively shown in Fig. 8b, c). Similarly, we identified 8400 differentially-expressed genes on comparing groups A and D (P-adjust < 0.05). There were 1757 up-regulated genes and 6643 down-regulated genes (Fig. 8d). The GO and KEGG pathway analysis were conducted as described above (Fig. 8e, f), and detailed results are shown in Additional file 9: Table S4 and Additional file 10: Table S5.

Unsurprisingly, a comparison of group A and E showed a similar trend with other groups. A total of 5873 genes were differentially expressed on comparing groups A and E (P-adjust < 0.05), and included 3373 up-regulated genes and 2500 down-regulated genes (Fig. 8g). The GO enrichment classification details are illustrated in Fig. 8h. Two molecular function terms were significantly enriched (Fig. 8h, blue column). Details of all the significant GO enrichment terms are shown (Additional file 11: Table S6). KEGG analysis showed that the “pathway in cancer” was significantly enriched (Fig. 8i), which demonstrated that the combination of FTS and SDT modulated cancer-related pathways. Details are shown in Additional file 12: Table S7.

## Discussion

For the sake of overcoming critical issues of traditional cancer treatment strategies, great efforts have been devoted to various non-invasive therapeutics, such as HIFU [46, 47], PDT [48, 49], and SDT [50, 51]. SDT was highlighted to effectively induce cancer-cell death and suppress tumor growth in diverse preclinical tumor models, such as breast cancer [52], brain glioma [53] and pancreatic cancer [54], and even in other clinical reports [55, 56]. The combination of IR820@NCP nano-sono-sensitizers and FTS killed cancer cells by inducing apoptosis by a further undefined biological mechanism. To our knowledge, this study has for the first time assessed the effect of FTS and SDT on cytotoxicity in HCC. The scRNA-Seq results showed that FTS and SDT significantly intervened in the heterogeneity of HCC and enhanced the extrinsic and intrinsic apoptotic pathways, thus exhibiting a synergistic anti-cancer effect.

First, a type of novel nanocapsule was synthesized to deliver nano-sono-sensitizers (Fig. 1a). Due to their nanoscale diameter (Fig. 1b), the nanocapsules were used as nuclei for cavitation effects with SDT. Combined treatment of SDT with nanocapsules and FTS achieved an encouraging apoptotic rate at approximately 80% (Fig. 2e). Moreover, IR820 nanocapsules did not cause any obvious apoptosis without LIFU irradiation (Fig. 2b).

Secondly, FTS significantly enhanced SDT-induced ROS generation and loss of MMP. As shown in Fig. 3a, obvious green fluorescence was clearly observed after treatment, which suggested that FTS and SDT could induce ROS generation. However, after FTS treatment the cells showed less green fluorescence as compared to other treatment groups. Group E showed the strongest fluorescence among all groups. By contrast, MMP analysis showed similar results with ROS generation. After FTS-treatment, the cells showed pale green fluorescence. By contrast, FTS pretreatment combined with SDT potently enhanced the green fluorescence of cells, indicating that SDT significantly enhanced FTS-induced MMP loss.

The multi-wavelength fluorescence study was designed to detect the targeted molecular imaging capacity of IR820@NCP. In this study, mice were injected with PBS, IR820, and IR820@NCP, respectively. Unsurprisingly, we detected fluorescence of IR820 in mice. Nevertheless, mice injected with pure IR820 solution showed sporadic fluorescence throughout the body (Fig. 4b). This might have been due to the IR820 solution lacking the ability to target tumors, which were easily metabolized during in vivo circulation. The area of the tumor in mice injected with IR820@NCP showed pole-strength fluorescence (Fig. 4c). The higher accumulation of IR820@NCP could benefit from the EPR effect. However, the maximum and minimum fluorescence intensities in each mouse were recorded. It was shown that there was no statistical difference in fluorescence intensity on comparing both groups (Fig. 4d; P = 0.4896). Consequently, nanocapsules might function effectively as a promising drug carrier, but cannot enhance fluorescence intensity.

Thirdly, we used scRNA-Seq to reveal potential therapeutic mechanisms and cellular heterogeneity of HCC after combined SDT and FTS treatment. We obtained single-cell transcriptome data of HCC xenografts before and after treatment. Cluster identity was defined by basing it on known cell-type markers and differentially expressed genes (Additional file 4: Figure S4 and Additional file 5: Figure S5). The cluster identity of single samples could not explain the mechanisms of the treatment methods. Hence, we employed integrative analysis of single-cell transcriptome data of three treatment groups and the negative control group.

The integrative single-cell analysis of Group A vs Group C showed that four typical variant clusters were identified (Fig. 5a, b). After FTS treatment, the hepatic stellate cell populations (cluster 6) and periportal portal endothelial cell populations (cluster 12) had significantly increased. This means that FTS up-regulated the expression of ACTA2 and CXCL14 in cluster 6 (Fig. 5d), and SEMA6D in cluster 12 (Fig. 5f). By contrast, interzonal portal endothelial cell populations (cluster 7) and typical epithelial cell populations (cluster 13) were reduced. In the case of cluster 13, this cluster disappears completely after FTS treatment. It could be demonstrated that FTS played an anti-tumor role by inhibiting epithelial-derived cells and down-regulating the expression of KRT6A, KRT19, NTS, and S100A9 (Fig. 5g).

By integrative single-cell analysis of Group A vs Group D, we identified four typical variant clusters (Fig. 6). The hepatic stellate cell populations (cluster 8) increased slightly by up-regulating the expression of ACTA2 and CXCL14 (Fig. 6f). Meanwhile, the typical epithelial cells did not survive SDT therapy, which was similar to that seen with Group A vs Group C (Fig. 6g). Conversely, immune cells decreased significantly after SDT treatment. It was reported that SDT-based cancer therapy modulated immune-related cells, and even enhanced immunotherapy [57]. This study provided additional evidence supporting the hypothesis that SDT-based cancer therapy is related to host immunity.

Inspired by the above results, the integrative single-cell analysis of Group A vs Group E was conducted. It turned out as expected that the interzonal portal endothelial cells (cluster 5), the immune-related cells (cluster 9), and portal periportal endothelial cells (cluster 10) were significantly reduced, while the hepatic stellate cells were increased significantly (Fig. 7a, b). Thus, we concluded that the combined treatment of SDT and FTS suppressed HCC, and did so mainly by inhibiting endothelial cells and modeling host immunity. By contrast, hepatic stellate cells secreted hepatocyte growth factor (HGF) and participated in the regulation of hepatocyte regeneration. This might represent another unidentified anti-tumor mechanism. After the analysis of scRNA-seq data, we found that CORO1A expression was down-regulated significantly in immune-related cells (cluster 9) after combined treatment (Fig. 7f).

To further study the roles and functions of differentially expressed genes, GO and KEGG enrichment analyses were conducted. According to the integrative enrichment analysis, most differentially expressed genes in Group A vs Group C and Group A vs Group D were mainly enriched in biological process terms (BP; Fig. 8b, e, as shown by the red column) and cellular component terms (CC, Fig. 8b, e, green column). After combined treatment, the enriched terms of molecular function (MF; Fig. 8e, as shown by the blue column) were significantly increased, especially in the context of “cadherin binding” and “cell adhesion molecule binding”. KEGG analysis revealed the pathways involved in differential gene expression (Additional file 12: Table S7). It is worth mentioning that the “pathway in cancer” was significantly enriched (Fig. 8i) after combined treatment, although this was not significantly enriched in Group A vs Group C and in Group A vs Group D. We inferred that the combined FTS and SDT treatment effectively regulated genes that played key roles in the “pathway in cancer” gene set.

## Conclusions

In conclusion, we report on the rational combination of nanocapsule-augmented SDT and a RAS inhibitor for highly efficient HCC therapy—a strategy that is based on the construction of a multi-functional nanocapsule (IR820@NCP). After systemic administration, IR820@NCP nano-sono-sensitizers achieve a degree of high tumor accumulation and fluorescence imaging, thereby effectively inducing tumor-cell death with contributions provided by non-invasive US irradiation. By employing scRNA-seq, we have demonstrated cellular heterogeneity in HCC after various treatments. Data presented in this study have provided us with a comprehensive understanding of the mechanism of our combined anti-tumor therapeutic strategy. Considering that SDT-based cancer therapy has already been used in the clinic, after further clarifying the mechanism of successful treatment with the scRNA-seq method, SDT might be a promising therapeutic option for treating cancer.

## Methods

### Preparation of IR820 nanocapsule sonosensitizers

IR820 nanocapsule (IR820@NCP) was prepared according to a typical reverse evaporation method [58, 59]. Briefly, IR820 was firstly dissolved in DMSO (10 mM). The phospholipid NBs were prepared from DSPG, DSPC, and DSPE-PEG2000 (Avanti Polar Lipids, Alabaster, AL, USA) with weight ratios of 7:2:1. Next, 20 mg of lipid powder mixture was dissolved in chloroform, 100 ul IR820 solution was added and then mixed. The following was evaporated on a rotary evaporator at 40 °C for 1 h to purge the chloroform and to obtain the dry lipid thin film on the bottle wall. After that, 5 ml of H_2_O was used to hydrate the lipid film, and then vortexed. The IR820 nanocapsules were obtained by an extrusion technology by mini-extruders (Avanti Polar Lipids, Alabaster, AL, USA) through a 400-nm membrane for 11 times at least. Ultimately, the IR820@NCP solution was stored at 4 °C for further experiments.

The structure of IR820@NCP was detected under the transmission electron microscope (TEM, Hitachi TEM system, Japan). The size and zeta potential of IR820@NCP were investigated by dynamic light scattering (DLS) via the Malvern Zetasizer Nanoseries (Zeta PALS BI-90 Plus, Brookhaven Instruments). The stability of the IR820 nanocapsule was evaluated by a hemocytometer at different time points, such as 6 h, 12 h, 24 h, and 48 h after preparation at 4 ℃.

### In vitro study

#### Cell culture

The HepG2 cells, a human HCC cell line, were a generous gift from the Institute of Cancer Prevention and Treatment with Heilongjiang Academy of Medical Science, (Harbin, China). Cells were cultured in DMEM medium supplemented with 10% fetal bovine serum and 1% penicillin/streptomycin in a humidified atmosphere containing 5% CO_2_/95% air at 37 °C.

#### Assay of cell cytotoxicity

Cell viability was studied in cells cultured with various concentrations of FTS and IR820, the HepG2 cells were cultured in 96-well plates (2 × 10^3^ cells per well). After the cells were cultured for 12 h, the cells were treated with FTS (50 to 225 μM) and IR820 (1 to 15 μM) for up to 24 h. Cell viability was detected by the CCK-8 kit according to the manufacturer's protocol. Cell viability was calculated by the following equation:

$${\text{Cell viability}}\left( \% \right)\, = \,{1}\, - \,\left[ {\left( {{\text{As}}\, - \,{\text{Ab}}} \right)\left] / \right[\left( {{\text{Ac}}\, - \,{\text{Ab}}} \right)} \right]{*1}00\%$$

As stands for the experimental group, Ab stands for the background group, and Ac stands for the control group. IC50 was calculated by nonlinear regression analysis using GraphPad Prism 8.0 software (San Diego, CA, USA).

#### In vitro cell apoptosis

To investigate the curative effect of sonodynamic therapy combined with RAS inhibitors, the HepG2 cells were divided into five groups: Group A: blank (PBS); Group B: IR820@NCP; Group C: FTS; Group D: IR820@NCP + SDT; Group E: IR820@NCP + SDT + FTS. After the cells were cultured for 12 h, the cells were treated with corresponding treatments. In the SDT group, US irradiation was conducted as 1.0 MHz, 1.0 W/cm^2^, 50% duty cycle, 1 min. Cell apoptosis detection was performed by flow cytometry (FCM, FACSCCanto II, BD Biosciences, New Jersey, USA) analysis using Annexin V-FITC/PI apoptosis detection kit (BD Biosciences, New Jersey, USA) according to the manufacture's protocol and 10,000 events were recorded in each sample. Apoptotic cells were those stained with Annexin V-FITC^+^/PI^−^ (early apoptotic cells) and Annexin V-FITC^+^/PI^+^ (late apoptotic cells). Results reflect the average of at least three replicates.

#### Detection of reactive oxygen species and mitochondrial membrane potential (MMP)

In brief, HepG2 cells were seeded in a 35-mm culture dish (2 × 10^5^ cells per dish) and cultured overnight. The cells were divided into five groups as described above. After 6 h following each treatment, the culture media was replaced by 100 μl DCFH-DA and fluorescent dye JC-1, respectively (1/9 μl in DMEM, Sigma-Aldrich, St Louis, MO, USA)). After incubating for another 30 min, the cells were washed with PBS three times and then observed by confocal laser scanning microscopy (CLSM), respectively. The quantitative analysis of ROS was conducted by flow cytometry.

### In vivo study

#### Animal tumor inoculation

Female BALB/c nude mice (4–6 weeks old and weighing 18–20 g) were purchased from Beijing Vital River Laboratory Animal Technology (Beijing, China). All the animals were housed at 22 ± 1 °C with a relative humidity of 50 ± 1%, and a light/dark cycle of 12:12 h. This study was approved by the Ethics Committee for Animal Experiments of Harbin Medical University. All animal experimental procedures (including the mice euthanasia procedure) were conducted according to the Association for Assessment and Accreditation of Laboratory Animal Care and the Institutional Animal Care and Use Committee guidelines.

For the establishment of the HCC tumor-bearing mouse model, mice were anesthetized with isoflurane inhalation. A total of 5 × 10^6^ HepG2 cells suspended in PBS (100 ul) was injected subcutaneously in the right-back flank.

#### Multiwavelength fluorescence imaging

Mice were randomly divided into six groups (n = 3), including (1) control, (2) only IR820, (3) IR820@NCP. The tumor diameter was allowed to reach 50 mm before the Imaging. All the therapeutic agents were intravenously administered via the tail vein at an equivalent IR-820 dose (1 mg/kg) body weight. The fluorescence intensity in mice was detected 1 h post-injection by the Kodak In Vivo Imaging Systems Fx Pro (Carestream Health, Rochester, NY, USA) using fluorescent excitation (790 nm) and emission filters (820 nm). The maximum intensity and minimum intensity in each mouse were recorded at the same time. Carestream Molecular Imaging Software v.5.0.2 (Carestream Health) was used for imaging and analysis. The major organs of mice, such as the heart, lung, spleen, and kidney, were harvested after euthanasia. The fluorescence intensity was detected as previously described.

#### Analysis of anti-tumor efficiency

Mice-bearing tumors were randomly divided into five groups (n = 6) as in vitro cell experiments. Once the tumor diameter reached 0.5 cm, all the therapeutic agents were intravenously administered via the tail vein at an equivalent IR-820 dose (1 mg/kg) and FTS dose (5 mg/kg). The nude mice were treated twice a week for a total of 30 days. In the SDT group, US irradiation (1.0 MHz, 1.0 W/cm^2^, 50% duty cycle, 3 min) was conducted 1 h post-injection. The survival endpoint was a tumor diameter of 20 mm in any direction (according to the Guidelines for Tumor Induction in Mice and Rats, American Association for Laboratory Animal Science, Memphis, TN, USA); the tumor diameter was measured every two days using Vernier caliper. After the end of the follow-up period, the remaining tumor samples were collected for IHC analysis.

A paraffin section of three samples of liver cancer tissue was prepared. Briefly, liver cancer tissue sections were deparaffinized and rehydrated. Subsequently, heat mediated antigen retrieved with EDTA (pH 9.0), then cooled for 30 min. The samples were quenched with 3% H_2_O_2_ for 10 min and blocked with goat serum for 10 min at room temperature. The sections were incubated overnight at 4 °C with primary antibodies: Caspase-3 (1:100). Following incubation with secondary antibody at 37 °C for 30 min. The sections were then stained with DAB and were counterstained with hematoxylin, dehydrated in alcohol, and mounted.

### Single-cell RNA sequencing analysis

On the 14th day, euthanasia of three tumor-bearing mice in Group A, Group C, Group D, and Group E, tumor samples were collected, dissected, and subjected to scRNA-seq.

#### Single-cell library preparation and sequencing

The cell suspension was loaded into Chromium microfluidic chips with 3’ v3 chemistry and barcoded with a 10× Chromium Controller (10× Genomics). RNA from the barcoded cells was subsequently reverse-transcribed and sequencing libraries constructed with reagents from a Chromium Single Cell 3’ v3 reagent kit (10× Genomics) according to the manufacturer’s instructions (https://support.10xgenomics.com/single-cell-gene-expression/sample-prep/doc/demonstrated-protocol-single-cell-protocols-cell-preparation-guide). Sequencing was performed with Illumina (HiSeq 2000) according to the manufacturer’s instructions Illumina.

#### Generation and analysis of single-cell transcriptomes

Raw reads were demultiplexed and mapped to the reference genome by 10× Genomics Cell Ranger pipeline (https://support.10xgenomics.com/single-cell-gene-expression/software/pipelines/latest/what-is-cell-ranger) using default parameters. All downstream single-cell analyses were performed using Cell Ranger and Seurat unless mentioned specifically [60, 61]. In brief, for each gene and each cell barcode (filtered by Cell Ranger), unique molecule identifiers were counted to construct digital expression matrices. Secondary filtration by Seurat: A gene with expression in more than three cells was considered as expressed, and each cell was required to have at least 200 expressed genes. And filter out some of the foreign cells.

#### Secondary analysis of gene expression

##### Seurat

The Seurat package was used to normalize data, dimensionality reduction, clustering, differential expression. we used Seurat alignment method canonical correlation analysis (CCA) for integrated analysis of datasets [62]. For clustering, highly variable genes were selected and the principal components based on those genes used to build a graph, which was segmented with a resolution of 0.6.

##### Global analysis between samples

Based on filtered gene expression matrix by Seurat, between samples differential expression analysis was carried out using the edgeR package to obtain zone-specific marker genes [63].

##### Enrichment analysis of marker genes

Gene Ontology (GO) enrichment analysis of marker genes was implemented by the cluster Profiler R package, in which gene length bias was corrected. GO terms with corrected P value less than 0.05 were considered significantly enriched by marker gene.

KEGG [64] is a database resource for understanding high-level functions and utilities of the biological system, such as the cell, the organism and the ecosystem, from molecular-level information, especially large-scale molecular datasets generated by genome sequencing and other high-through put experimental technologies (http://www.genome.jp/kegg/). We used cluster Profiler R package to test the statistical enrichment of marker genes in KEGG pathways.

### Statistical analysis

Statistical tests employed with the number of replicates and independent experiments are listed in the text and figure legends. All graphs with error bars report mean ± s.e.m. values except where indicated. One-way analysis of variance (ANOVA) with repeated measures was used to determine the significance of differences among the groups. GraphPad PRISM 8.0 was used for basic statistical analysis and plotting, and the R language and programming environment was used for the remainder of the statistical analysis.

## Supplementary Information

**Additional file 1: Figure S1.** Stability of the IR820 nanocapsule. The concentrations of IR820 nanocapsule at 0 h, 6 h, 12 h, 24 h and 48 h after preparing. No significant difference of concentration within 24 h. A slight decrease in concentration was observed after 24 h. Brown-Forsythe test shown that there was no statistical significance.

**Additional file 2:** **Figure S2.** Cytotoxicity analysis of IR820 and FTS. (a) Cell viability of HepG2 cells treated with IR820 at different concentrations. (b) Cell viability of HepG2 cells treated with FTS at different concentrations. (c) The dose–effect curves of HepG2 cells to IR820. (d) The dose–effect curves of HepG2 cells to FTS.

**Additional file 3: Figure S3.** Fluorescence of major organs. The fluorescence image of heart (a), lung (b) and spleen (c), there was nearly no fluorescence accumulation could not be detected. (d) However, the kidney showed faint fluorescence accumulation than other organs.

**Additional file 4: Figure S4.** Clustering of HCC xenograft single cells. Two-dimensional tSNE plot of tumor sample in Group A (a), in Group C(b), in Group D(c) and in Group E(d). Cells were colored according to cluster identity as shown in the Figure key.

**Additional file 5: Figure S5.** Heatmap of differentially expressed genes used to classify cell types for each cluster in Group A (a), in Group C(b), in Group D(c) and in Group E(d).

**Additional file 6: Table S1.** Sequencing information.

**Additional file 7: Table S2.** The GO enrichment terms of Group A vs Group C.

**Additional file 8:** **Table S3.** The KEGG enrichment terms of Group A vs Group C.

**Additional file 9: Table S4.** The GO enrichment terms of Group A vs Group D.

**Additional file 10:** **Table S5.** The KEGG enrichment terms of Group A vs Group D.

**Additional file 11**: **Table S6.** The GO enrichment terms of Group A vs Group E.

**Additional file 12: Table S7.** The KEGG enrichment terms of Group A vs Group E.

## Data Availability

The datasets used and analyzed during the current study were uploaded to a web repository, and available from the corresponding author on reasonable request.

## Abbreviations

HCC: Hepatocellular carcinoma
SDT: Sonodynamic therapy
FTS: Farnesyl-thiosalicylic acid
PDT: Photodynamic therapy
ROS: Reactive oxygen species
LIFU: Low intensity focused ultrasound
scRNA-seq: Single-cell RNA sequencing
MMP: Mitochondrial membrane potential
