# Supplementary figures and images for: Single-cell RNA sequencing reveals the mechanism of sonodynamic therapy combined with a RAS inhibitor in the setting of hepatocellular carcinoma
Source: J Nanobiotechnology. 2021 Jun 12;19:177. doi: 10.1186/s12951-021-00923-3 (PMC8199394; doi:10.1186/s12951-021-00923-3)

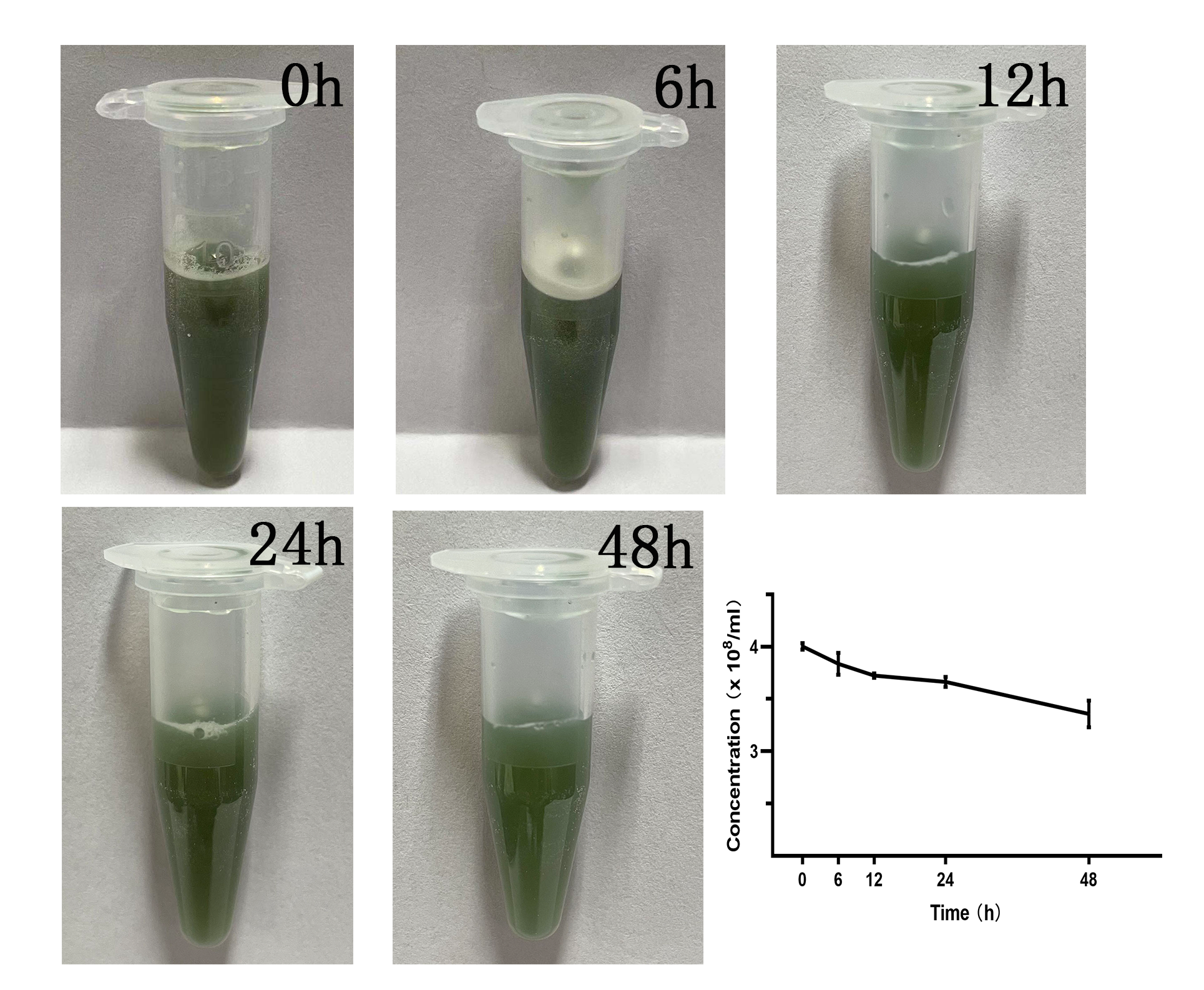

Supplement: Supplementary file 1 — Additional file 1: Figure S1. Stability of the IR820 nanocapsule. The concentrations of IR820 nanocapsule at 0 h, 6 h, 12 h, 24 h and 48 h after preparing. No significant difference of concentration within 24 h. A slight decrease in concentration was observed after 24 h. Brown-Forsythe test shown that there was no statistical significance. [file 12951_2021_923_MOESM1_ESM.tif]

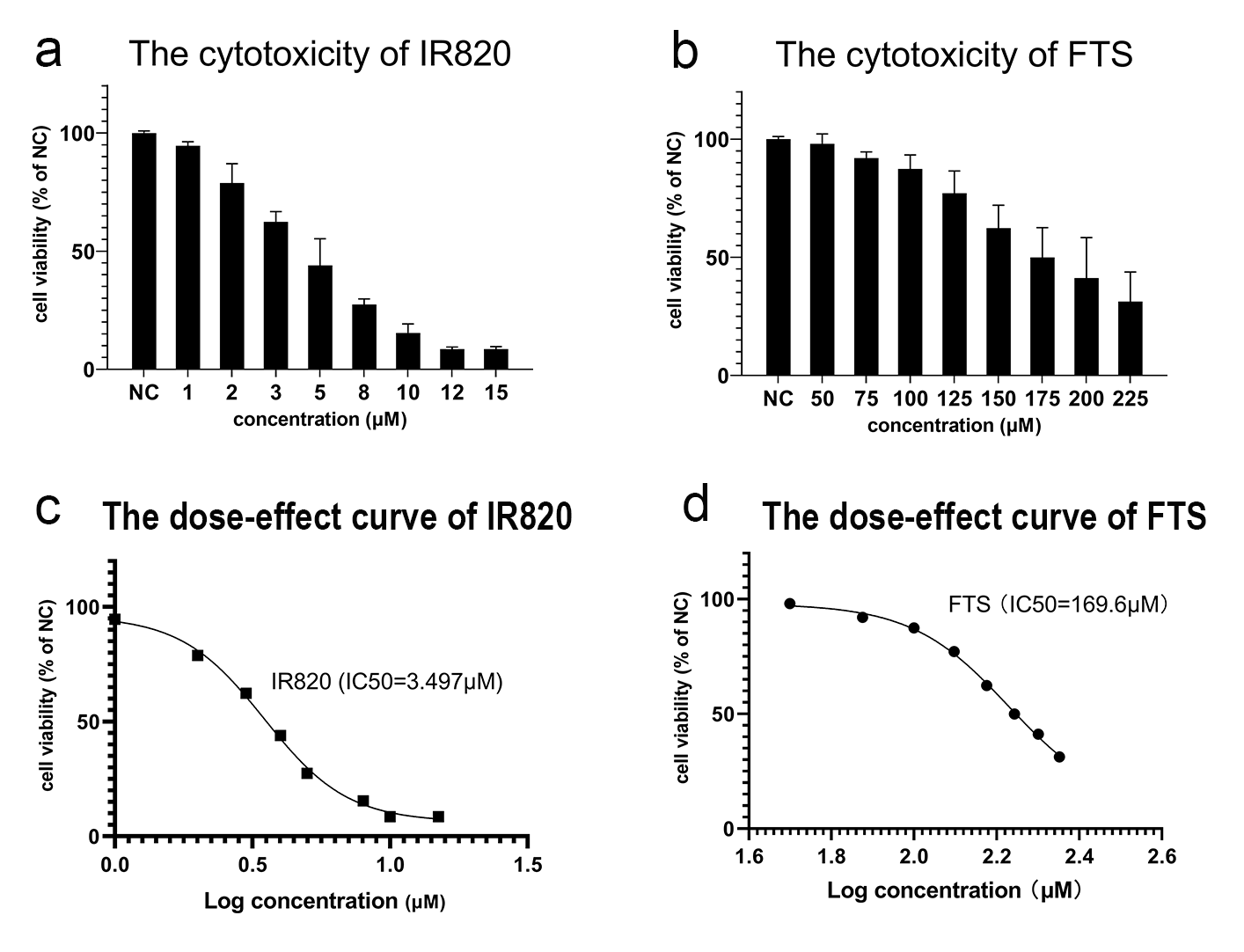

Supplement: Supplementary file 2 — Additional file 2: Figure S2. Cytotoxicity analysis of IR820 and FTS. (a) Cell viability of HepG2 cells treated with IR820 at different concentrations. (b) Cell viability of HepG2 cells treated with FTS at different concentrations. (c) The dose–effect curves of HepG2 cells to IR820. (d) The dose–effect curves of HepG2 cells to FTS. [file 12951_2021_923_MOESM2_ESM.tif]

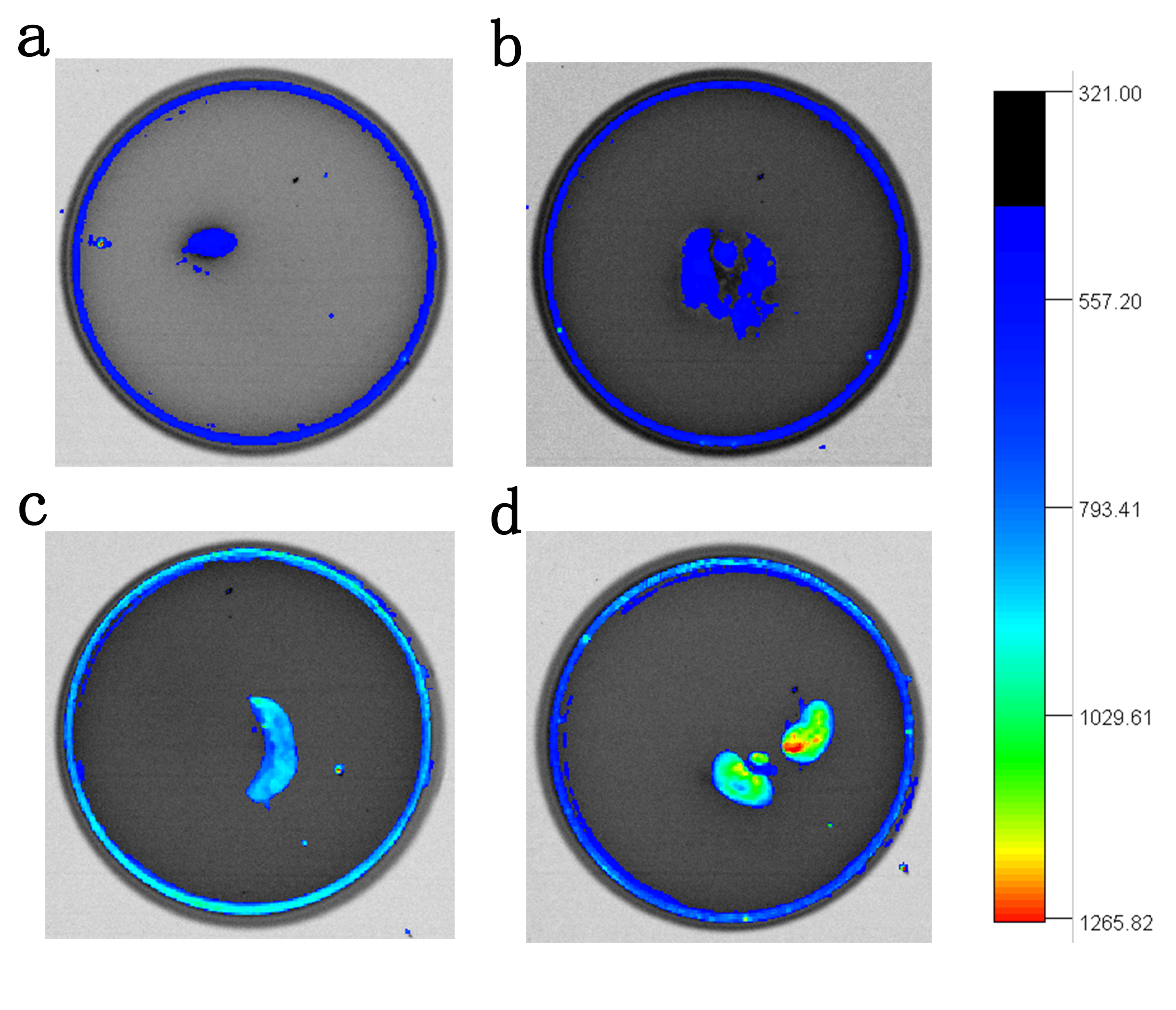

Supplement: Supplementary file 3 — Additional file 3: Figure S3. Fluorescence of major organs. The fluorescence image of heart (a), lung (b) and spleen (c), there was nearly no fluorescence accumulation could not be detected. (d) However, the kidney showed faint fluorescence accumulation than other organs. [file 12951_2021_923_MOESM3_ESM.tif]

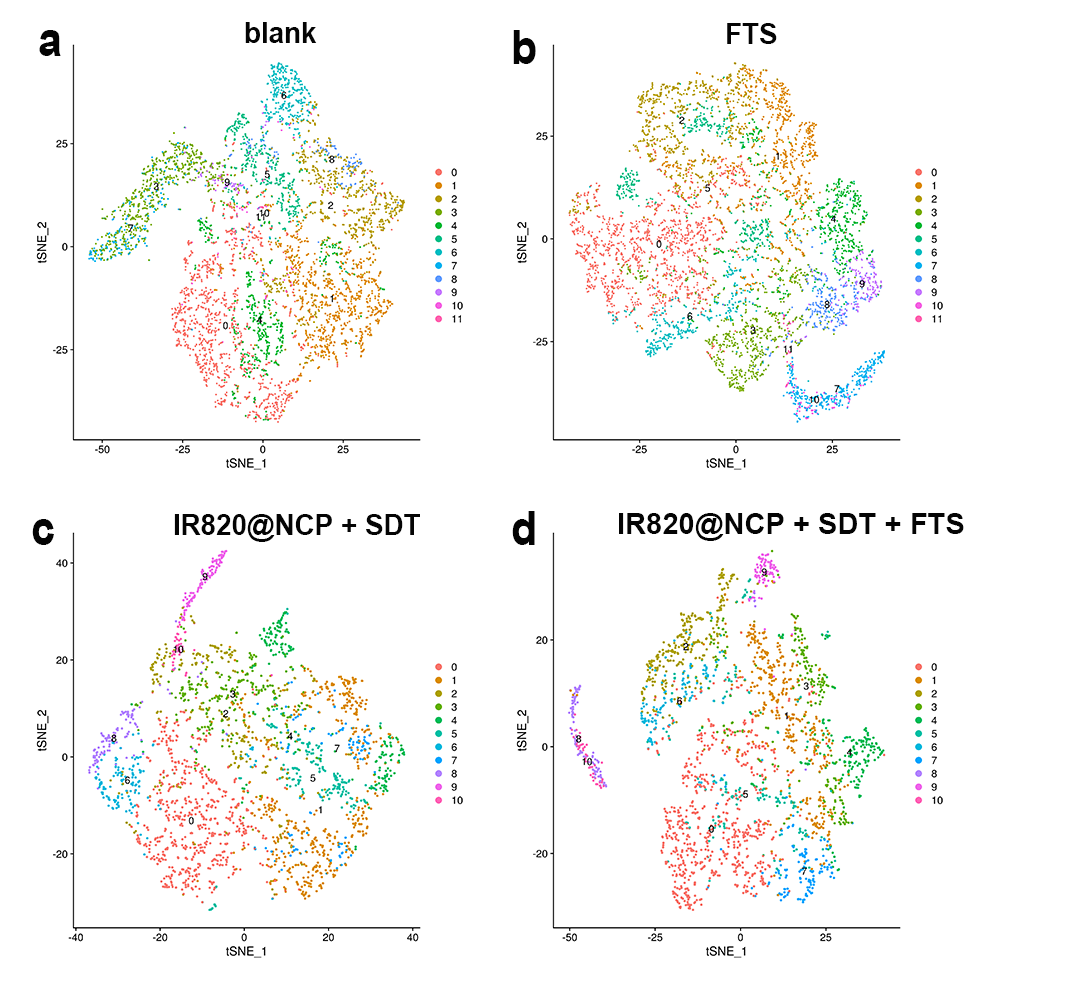

Supplement: Supplementary file 4 — Additional file 4: Figure S4. Clustering of HCC xenograft single cells. Two-dimensional tSNE plot of tumor sample in Group A (a), in Group C(b), in Group D(c) and in Group E(d). Cells were colored according to cluster identity as shown in the Figure key. [file 12951_2021_923_MOESM4_ESM.tif]

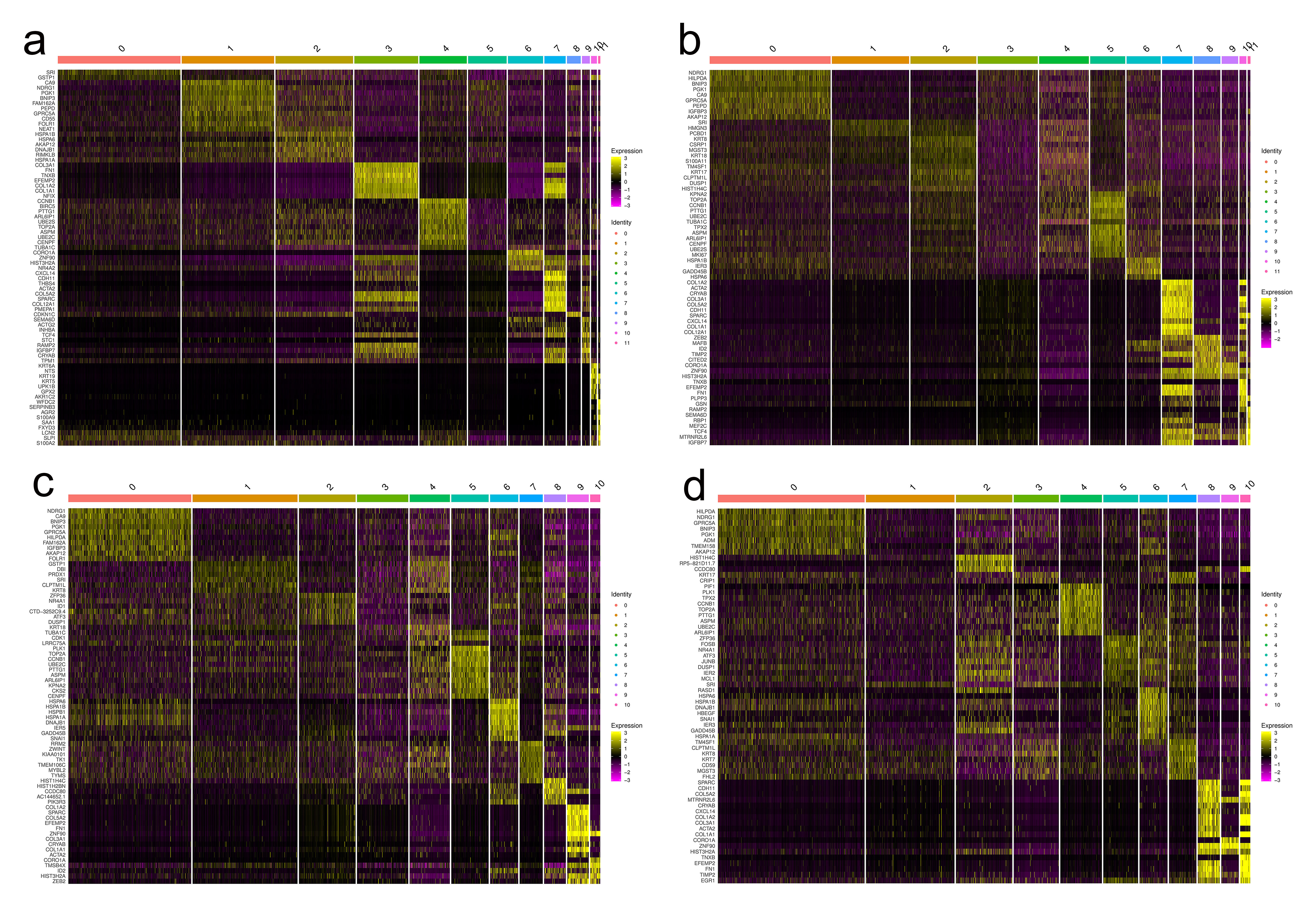

Supplement: Supplementary file 5 — Additional file 5: Figure S5. Heatmap of differentially expressed genes used to classify cell types for each cluster in Group A (a), in Group C(b), in Group D(c) and in Group E(d). [file 12951_2021_923_MOESM5_ESM.jpg]
