# Supplementary material for: Single-cell RNA sequencing reveals the mechanism of sonodynamic therapy combined with a RAS inhibitor in the setting of hepatocellular carcinoma
Source: J Nanobiotechnology. 2021 Jun 12;19:177. doi: 10.1186/s12951-021-00923-3 (PMC8199394; doi:10.1186/s12951-021-00923-3)
Supplement: Supplementary file 6 — Additional file 6: Table S1. Sequencing information. [file 12951_2021_923_MOESM6_ESM.docx]

| Sample | Total Reads | Number of Cells | Mean Reads per Cell | Total Genes Detected | Median Genes per Cell |
| --- | --- | --- | --- | --- | --- |
| Group A | 706,024,986 | 7,283 | 96,942 | 22,921 | 1,674 |
| Group C | 614,150,300 | 7,610 | 80,703 | 22,458 | 2,380 |
| Group D | 692,336,247 | 3,724 | 185,912 | 21,612 | 3,207 |
| Group E | 666,216,57 | 3,103 | 214,701 | 22,090 | 3,019 |

Table S1 Sequencing information
